# Carcinoid tumor of the kidney: An unusual renal tumor

**DOI:** 10.4103/0970-1591.57921

**Published:** 2009

**Authors:** P. P. Singh, Amit Singh Malhotra, Vikas Kashyap

**Affiliations:** Batra Hospital and Medical Research Centre, 1, Tughlakabad institutional area, M.B. Road, New Delhi -110 062, India

**Keywords:** An unusual renal tumor

## Abstract

Carcinoid tumors are low-grade malignant tumors that arise from neuroendocrine cells. Primary renal carcinoid is extremely rare. We present a case of 57-year-old male with primary renal carcinoid tumor. Presently, the patient is on regular follow up and is doing well.

## INTRODUCTION

Carcinoid tumors most commonly arise in lung and gastrointestinal tract, but less frequently in kidneys, breast, ovaries, testis, prostate, and other locations. The pathogenesis is uncertain because neuroendocrine cells are not found in renal parenchyma, pelvis, and ureter. Only 43 cases of primary renal carcinoid tumor (RCT) have been reported so far, of these only six had characteristic carcinoid syndrome. We hereby report a case of RCT and provide a review of the literature.

## CASE REPORT

**Figure 1:**
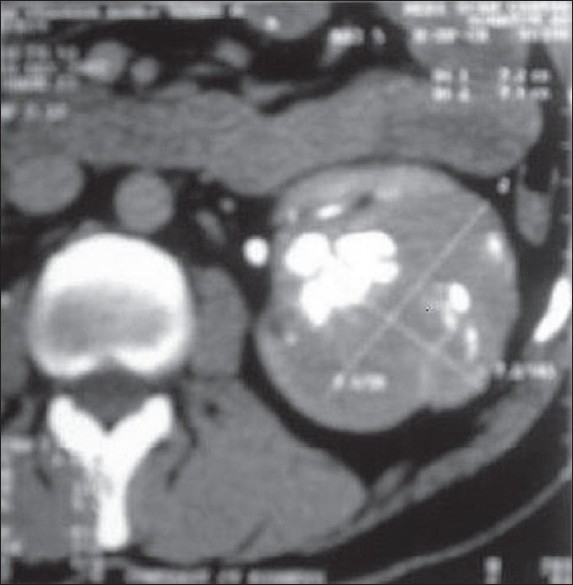
Computed tomographic scan of abdomen demonstrating left lower pole renal mass with areas of calcification

**Figure 2:**
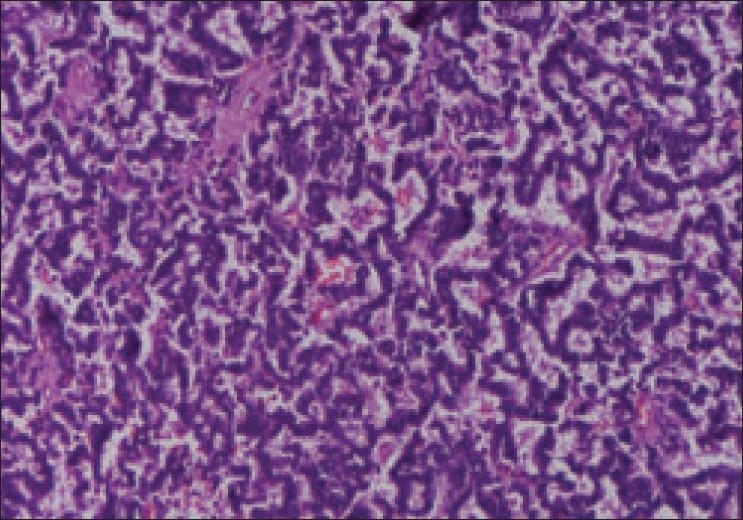
Microscopy revealing classical carcinoid organoid and tubulopapillary pattern with focal rosettes and delicate fibrovascular stroma

A 57-year-old male with painless hematuria and normal physical examination was found to be having left renal mass on ultrasound. He had no symptoms suggestive of carcinoid syndrome like headache, flushing, palpitations, or diarrhea. X-ray of the kidney and urinary bladder revealed calcification in left renal area. CECT [Figure 1] revealed left lower pole mass with areas of calcification. Bone scan was normal. The patient underwent left radical nephrectomy. Enlarged hilar and para-aortic lymph nodes were sampled. A 8.5 × 7.5 × 7.5 cm grayish-white tumor with chalky white areas of calcification was present extending from renal pelvis to capsule with adherent perinephric fat. Microscopy [Figure 2] revealed classical carcinoid showing organoid and tubulopapilary pattern with focal rosettes and delicate fibrovascular stroma. Large areas of calcification and psammoma bodies were seen. Tumor cells were monomorphic with stippled chromatin pattern. Two para-aortic lymph nodes showed metastatic tumor deposits with solid growth pattern. Immunohistochemistry for synaptophysin and chromogranin was positive. Twenty four hour urinary 5-HIAA was normal. Somatostatin receptor (SR) scintigraphy octreotide scan done a month later was unremarkable. CT scan done after a year of the surgery did not reveal any evidence of recurrence.

## DISCUSSION

The pathogenesis of RCT is controversial. Primary RCTs are probably derived from interspersed neuroendocrine cells associated with acquired and congenital abnormalities such as metaplasia of pelvicalyceal urothelium induced by chronic inflammation, misplaced or entrapped neural crests or pancreatic tissue in kidney during embryogenesis, activation of gene sequences common to neuroendocrine programed cells in multipotent stem cells, or concurrent congenital abnormalities.[1] The mean age at presentation is 50, with predilection for either sex. Most patients present with abdominal pain and hematuria, and majority are diagnosed following surgical excision of tumor, only 13.6% patients have features of carcinoid syndrome at presentation. Although primary RCTs appear to be more indolent than renal cell carcinomas, metastasis to the regional lymph nodes, liver, lungs, and bones have been described often with malignant course. Typically these are solid tumors with occasional cystic components with significant necrosis and dystrophic calcification. No specific feature differentiates RCTs from other renal tumors on CT and MRI. The differential diagnosis on routine histology includes neuroendocrine carcinoma, primitive neuroectodermal tumor, neuroblastoma, and Ewings sarcoma. Histochemical staining (Argentaffin stain) and immunohistochemical positivity of tumor cells for neuroendocrine markers such as chromogranin, synaptophysin, neuron specific enolase, and leu-7[2] confirms the diagnosis. Surgical excision is the mainstay of treatment.[3] Nephron sparing surgery is preferred if the diagnosis is suspected preoperatively. Whenever a carcinoid tumor is found within the kidney, the need for a thorough search for an unknown primary tumor is indicated. SR scintigraphy is recognized as an integral diagnostic and staging tool in the evaluation of carcinoid tumors, because CT and MRI occasionally lack sensitivity in detecting these tumors. Preoperative octreotide scan is reserved for patients who present with clinical history that is suspicious of neuroendocrine tumor supported by elevated levels of primary hormones and/or metabolites in plasma and/or urine. Radiolabeled octreotide somatostatin analog that binds SR with high affinity, is useful in detecting even the smallest carcinoid tumors with 85% sensitivity.[4] The octreotide scan is also important investigation for surveillance. CT and/or MRI can also be used as imaging studies for surveillance. The treatment of liver metastasis from RCT is not well defined. Most of the experience regarding the treatment of metastatic neuroendocrine disease in the liver comes from those tumors originating in the gastrointestinal tract, and in these cases the mainstay of treatment is resection.[3] In the last few years some authors have suggested that even in the presence of extensive disease, liver resection for cytoreduction may be not only palliative but also may increase survival. Nagorney *et al.* have proposed that surgical resection is indicated if the primary lesion is resectable or has been resected, which makes 90% of liver metastasis either resectable or ablatable. An impressive four-year survival rate of 75% has been achieved with this approach. Cytotoxic chemotherapy has only limited success in treatment of metastatic carcinoid tumors. Long-term follow up is recommended as metastasis can occur years after resection. Resection, ablation, or both in combination can be used to treat tumor recurrences. Somatostatin analogs, like octreotide and more recently lanreotide, which can be given monthly, have been utilized to treat patients with advanced disease[5] with a response rate of 36–70%. Interferon alpha has been used in neuroendocrine tumors with low response rate, but stabilization of disease has been observed in 40–60% of cases.

## CONCLUSION

Early surgical intervention together with careful surveillance and follow up can achieve successful long-term outcomes in patients with this rare malignancy.
